# Conservation of Helical Bundle Structure between the Exocyst Subunits

**DOI:** 10.1371/journal.pone.0004443

**Published:** 2009-02-13

**Authors:** Nicole J. Croteau, Melonnie L. M. Furgason, Damien Devos, Mary Munson

**Affiliations:** 1 Department of Biochemistry and Molecular Pharmacology, University of Massachusetts Medical School, Worcester, Massachusetts, United States of America; 2 EMBL, Heidelberg, Germany; Cardiff University, United Kingdom

## Abstract

**Background:**

The exocyst is a large hetero-octomeric protein complex required for regulating the targeting and fusion of secretory vesicles to the plasma membrane in eukaryotic cells. Although the sequence identity between the eight different exocyst subunits is less than 10%, structures of domains of four of the subunits revealed a similar helical bundle topology. Characterization of several of these subunits has been hindered by lack of soluble protein for biochemical and structural studies.

**Methodology/Principal Findings:**

Using advanced hidden Markov models combined with secondary structure predictions, we detect significant sequence similarity between each of the exocyst subunits, indicating that they all contain helical bundle structures. We corroborate these remote homology predictions by identifying and purifying a predicted domain of yeast Sec10p, a previously insoluble exocyst subunit. This domain is soluble and folded with approximately 60% α-helicity, in agreement with our predictions, and capable of interacting with several known Sec10p binding partners.

**Conclusions/Significance:**

Although all eight of the exocyst subunits had been suggested to be composed of similar helical bundles, this has now been validated by our hidden Markov model structure predictions. In addition, these predictions identified protein domains within the exocyst subunits, resulting in creation and characterization of a soluble, folded domain of Sec10p.

## Introduction

The exocyst is a large, eight protein complex localized to sites of polarized secretion that is required for exocytosis and cytokinesis in eukaryotes ([1]–[3]; and references therein). Its specific function(s) is unclear, but it interacts with small Ras superfamily GTPases on secretory vesicles and the plasma membrane, where it is hypothesized to tether vesicles to the plasma membrane prior to membrane fusion [4]. The complex also interacts with the regulatory protein, Sec1p [5], and the plasma membrane SNARE (soluble N-ethylmaleimide sensitive protein receptor) protein Sec9p [6]. These interactions indicate a role for the exocyst and its subunits in the quality control of exocytic trafficking, as well as in facilitating SNARE complex assembly and vesicle fusion at the plasma membrane. Elucidation of the exocyst's function, and that of other related tethering complexes [7]–[10], requires biochemical and structural analyses of the individual subunits, as well as various protein-protein interactions within the complex.

**Figure 1:**
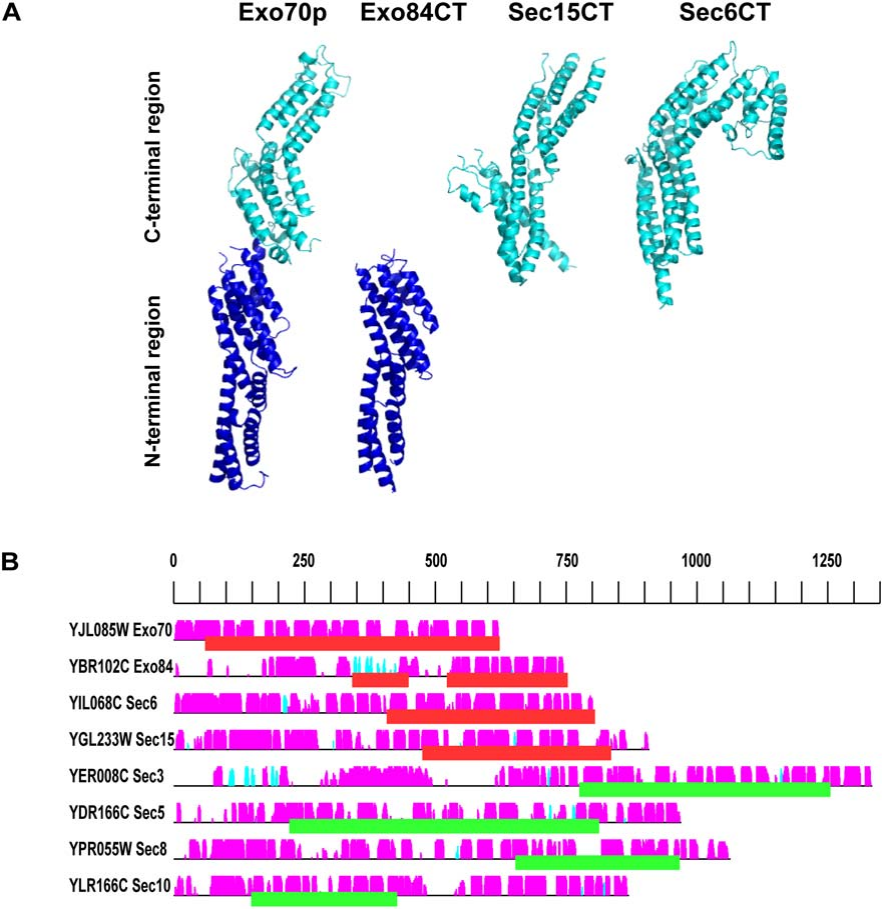
The exocyst subunits have similar helical bundle structures. (*A*) The known structures of the exocyst subunits are shown: Exo70p (PDB ID 2B1E), Exo84CT (PDB ID 2D2S), Sec15CT (PDB ID 2A2F), Sec6CT (PDB ID 2FJI). Molecular graphics were generated with PyMOL (http://pymol.sourceforge.net/). Exo84CT is aligned with the N-terminal helical bundles of Exo70p, while Sec15CT and Sec6CT are aligned with the C-terminal bundles of Exo70p. (*B*) Secondary structure predictions for all of the exocyst subunits. The black horizontal lines represent the sequence of each yeast exocyst subunit. The predicted α-helices (magenta) and β-strands (cyan) are indicated by vertical bars above each line. The height of the bars is proportional to the confidence of the secondary structure prediction [41]. Red blocks underline regions of the known structures. Green blocks underline the best hits to exocyst structures (see Table 1).

Each of the exocyst subunits is predicted to be α-helical. They possess less than 10% sequence identity with each other, although limited sequence similarity has been detected using PSI-BLAST analyses [7], [11]. They also show little similarity to other proteins or domains, except for short regions of predicted coiled coils [1], [7]. Several recent crystal structures of domains from individual subunits have been determined: nearly full-length yeast and human Exo70 [12]–[14], and the C-terminal domains of yeast Exo84p [12], yeast Sec6p [15] and *Drosophila* Sec15 [16]. They show similar structures containing multiple helical bundles, yielding an overall similar shape (Figure 1A). Specific details of the bundles differ, especially the surface residues, but the helical bundle topologies are identical, suggesting divergent evolution from an ancient exocyst ancestor protein for these four exocyst components [3], [15].

Progress for the other subunits has been hindered by lack of soluble protein. Insolubility can occur for many different reasons: recombinant proteins may not fold correctly when overexpressed in *Escherichia coli* cells, they may not have the correct post-translational modifications, or they may be insoluble in the absence of co-factors or binding partners. Much effort has been spent to develop methods to address these issues ([17]–[21]; and references therein), including the use of different strains, selective growth conditions, fusion tags, co-expression with binding partners, and expression of independently folded structural domains. Often, the latter strategy is approached by using secondary structure predictions to deduce a domain, making numerous constructs with slight variations at the N- and/or C-termini, or by using limited proteolytic digestions to cleave unstructured or floppy regions, thereby defining a core domain. Limited proteolysis proved critical for most of the current exocyst structures; however, this strategy relies on availability of at least slightly soluble protein. An alternative approach is to computationally predict domain structures based on the similarity to proteins with known structural domains [20], [22]–[24]. However, this approach is challenging if the protein has little or no similarity with proteins of known structures. In that case, more sensitive computational methods, such as hidden Markov model (HMM) predictions [25], may be successful.

Based on the structural conservation observed in the exocyst subunits, we hypothesized that the other subunits would have similar helical bundle structures [3]. Therefore, we examined the conservation of structural similarity between the subunits by profile HMM analyses using the HHSearch program [26]. Profile HMMs are similar to simpler sequence profiles, but in addition to the amino acid frequencies in the columns of a multiple sequence alignment, they contain information about the frequency of inserts and deletions at each column, plus transition probabilities. In addition, secondary structure can be included in the HMM-HMM comparison, leading to another increase in sensitivity. We applied state-of-the-art HMM-HMM comparisons to the exocyst complex and detected structural similarity between all of the exocyst subunits.

We verified these structure predictions by identifying a structural domain in one of the exocyst subunits, Sec10p (YLR166C), from *Saccharomyces cerevisiae*. Sec10p is one of the core subunits in the complex and has previously been shown to interact *in vitro* with its partner exocyst subunits Sec6p, Exo70p and Sec15p [2], [12], [15]. The specific function(s) of Sec10p is unknown; however, overexpression of N- and C-terminal truncated constructs show dominant negative secretory and morphogenic defects *in vivo* [27]. Further characterization of Sec10p by biophysical and structural methods has been hindered by the lack of soluble recombinant Sec10p protein. We expressed and purified the predicted Sec10p structural domain, and show that it is folded and helical in solution. This domain is functional—it retains the ability to interact with both Sec6p and Exo70p. In addition, we show that it interacts directly with the C-terminal domain of Exo84p, an interaction previously shown only by yeast two-hybrid studies [28]. Thus, our bioinformatic analyses have revealed structural similarity between all exocyst components and have additionally defined a soluble domain of the exocyst complex subunit Sec10p for further biochemical and structural characterization.

## Results and Discussion

### All of the exocyst subunits have similar helical bundle structures

The exocyst complex is composed of eight large proteins (between 71 and 155 kD) that are predicted to be predominantly helical by secondary structure predictions (Figure 1B). They form a complex at sites of exocytosis, have been proposed to tether secretory vesicles to the plasma membrane, and may serve as a quality control mechanism to ensure proper membrane fusion [3], [4]. Little is known about the structure of the intact complex, except a series of images taken by quick freeze-deep etch EM [29]. With less than 10% sequence identity between them, the exocyst subunits were originally thought to be unrelated. However, several short stretches in each of the subunits are predicted to form coiled coil (or amphipathic helical) structures [1], [7], [30], and regions of similarity with subunits from other tethering complexes have been detected [7], [11], [30]. When the high resolution crystal structures of several exocyst subunits (Sec15p, Exo70p, Exo84CT, and Sec6CT) were determined, it became clear that they are structurally and topologically similar (Figure 1A; [3]). Searches of the Protein Data Bank (PDB; [31] using Dali (http://www.ebi.ac.uk/dali) indicated that these helical bundle structures are considerably more similar to each other than to other proteins. The structures of the cargo binding domain of Myo2p, the unconventional type V myosin that transports yeast secretory vesicles [32], as well as the Conserved Oligomeric Golgi tethering complex subunit, COG2 (residues 61–262; [9]), also show structural similarity to the exocyst helical bundles. It is unclear whether the similarity of COG2 represents divergence or functional convergence of the COG complex subunits from the exocyst subunits [7], [9], [11], [30]. The structural relatedness between the different exocyst subunits, combined with similar patterns of predicted helical secondary structures (Figure 1; also [12], [15], [16]) led us to predict that all the exocyst structures would be similar, and to create a new working model for the exocyst complex structure [3]. This structural similarity was recently supported using multiple iterations of PSI-BLAST [33], for four of the exocyst subunits [11].

Here, we use hidden Markov models to examine the relatedness of the exocyst subunits at the sequence level. These analyses use comparisons of different HMM profiles generated from the individual exocyst families combined with secondary structure predictions and the known structures; this method has previously been shown to be more sensitive than other current analyses [26]. Indeed, statistically significant P-values were detected between all the exocyst components of known structure (Table 1). Two families of exocyst structures can be distinguished in the helical bundle structures of the exocyst components [not including the Ral binding domains of mammalian Sec5 [34], [35] and Exo84 [36]]: the Exo70/84 and Sec6/15 families. The Exo70 (2B1E_A & 2PFT_A) HMM detects the Exo84 (2D2S_A) HMM with a P-value of 10^−4^ and reciprocally, while the Sec6 (2FJI_1) and Sec15 (2A2F_X) HMMs detect each other with 10^−3^ and 10^−7^ P-values. All exocyst sequences of unknown structures can be linked to at least one exocyst sequence of known structure (Table 1).

**Table 1:** Similarity to exocyst subunits of known structures.

		Length(aa)	scExo70p	musExo70	Exo84CT	Sec6CT	dmSec15CT
			2b1e_A	2pft_A	2d2s_A	2fji_1	2a2f_X
YJL085W	**Exo70p**	622	**0** (60–623)	**0** (70–622)	**2.1E-04** (94–288)	**1.7E-01** (12–342)	**6.2E-03** (241–383)
YBR102C	**Exo84p**	752	**3.8E-03** (549–747)	**8.5E-04** (549–707)	**0** (523–753)		
YIL068C	**Sec6p**	804	**3.0E-01** (98–666)	**5.1E-01** (361–802)	**2.5E-01** (174–385)	**0** (407–805)	**4.8E-07** (413–728)
YGL233	**Sec15p**	909	**2.6E-02** (159–684)	**2.7E-01** (432–746)	**4.6E-02** (81–252)	**4.2E-03** (491–861)	**0** (475–836)
YER008C	**Sec3p**	970	**1.0E-07** (720–1333)	**2.6E-08** (733–1329)		**4.7E-03** (1046–1305)	
YDR166C	**Sec5p**	1335	**9.4E-01** (221–660)	**4.4E-01** (153–551)	**2.2E-06** (228–378)	**2.1E-04** (687–937)	**4.8E-08** (625–920)
YPR055W	**Sec8p**	870	**1.3E-01** (157–686)	**7.7E-02** (404–677)	**1.4E-05** (161–228)	**7.7E-03** (590–997)	**3.6E-07** (864–967)
YLR166C	**Sec10p**	1064	**1.3E-02** (187–695)	**3.1E-03** (186–465)	**1.7E-03** (178–393)	**2.0E-04** (563–860)	**1.4E-06** (578–826)

HMM P-values of the comparisons are shown in bold, with the range of the aligned residues in parentheses below. Yeast protein lengths are indicated by the number of amino acids (aa). SGD identifiers are indicated in the first column of the table, and PDB identifiers are indicated in the second row of the table. Blank cells have P-values>1.

The exocyst subunits show a striking pattern with many of their N-terminal regions detecting the Exo70/84 families, and many of their C-terminal regions detecting the Sec6/15 families (Table 1). The exceptions are Exo84 and Sec3, which differ in their N-terminal regions; both contain coiled coil and β-sheet domains. The similarities suggest that the Sec3/5/8/10 proteins are formed by tinkering with modules based on the exocyst families of known structures: N-terminal modules are derived from the Exo70/84 families and C-terminal modules from the Sec6/15 families. These analyses suggest an ancient gene duplication event, followed by fusion of these genes and divergence, then followed by multiple gene duplication events and divergence to create the different subunits. Additional modules, such as the coiled coil and β-sheet regions of Sec3p and Exo84p and the Ral-binding domains of the mammalian Sec5 and Exo84 proteins [34]–[36] were perhaps later acquisitions.

The similarity of the N-terminal modules is intriguing. With the exception of Exo70p, the N-terminal domains have not been soluble enough for biophysical and structural studies. They may be unstable, sticky, or perhaps natively unfolded in the absence of other exocyst subunits. It is tempting to speculate that the N-terminal domains might participate in protein-protein interactions at the core of the assembled exocyst, as seen in the electron micrographs of the bovine exocyst complex [29]. The C-terminal domains, therefore, might then play roles in interactions with small GTPases, the membrane, and other potential binding partners [3], [4].

Because the exocyst structures comprise only domains of the proteins, we repeated the HMM predictions with the full-length sequences. We determined that all the exocyst subunits can be linked to each other (Table 2). The difference in P-values between Table 1 and Table 2 is due to the usage of the complete sequence versus only parts of it. These results corroborate the preliminary analyses by [11], implying that all members of the exocyst complex are sequence related, and indicating that all exocyst subunits have a related structure.

**Table 2:** Similarity between full-length exocyst subunits.

		Exo70	Exo84	Sec6	Sec15	Sec3	Sec5	Sec8	Sec10
YJL085W	**Exo70p**	0	4.40E-02	1.20E-01	2.30E-02	6.30E-05	1.30E-01	4.50E-02	6.70E-07
YBR102C	**Exo84p**	4.60E-02	0	2.00E-02	2.50E-02	1.00E-02	2.10E-06	3.80E-04	4.40E-03
YIL068C	**Sec6p**	1.20E-01	1.90E-02	0	8.60E-02	5.70E-06	1.40E-02	5.80E-02	8.50E-03
YGL233W	**Sec15p**	2.30E-02	2.40E-02	8.80E-02	0	1.40E-02	2.10E-07	1.00E-08	3.60E-04
YER008C	**Sec3p**	9.10E-05	1.10E-02	1.10E-05	1.60E-02	0	2.40E-03	6.60E-04	1.40E-05
YDR166C	**Sec5p**	1.40E-01	3.80E-06	1.60E-02	4.90E-07	2.40E-03	0	1.10E-05	5.20E-05
YPR055W	**Sec8p**	4.10E-02	2.90E-04	5.40E-02	6.40E-09	3.60E-04	4.40E-06	0	9.30E-03
YLR166C	**Sec10p**	2.10E-06	6.90E-03	1.30E-02	7.70E-04	2.30E-05	8.60E-05	1.60E-02	0

HMM P-values of the comparisons are indicated for the full length proteins. SGD identifiers are indicated in the first column of the table.

### Creation and purification of a soluble yeast Sec10p construct

In order to understand more about the structure and function of the exocyst complex, it is imperative to have soluble purified subunits for structural studies and for reconstitution of the functional complex *in vitro*. Identification of a soluble structural domain for a previous insoluble exocyst subunit would validate our structure predictions, as well as provide soluble protein for further characterization. We chose to examine a predicted domain of the yeast Sec10p. Sec10 is one of the central subunits in the exocyst complex, and qualitative binding studies and yeast two-hybrid analyses indicate that it interacts with other exocyst subunits: Sec5, Sec6, Sec8, Sec15, Exo70 and Exo84 [3], [12], [15]. In addition, overexpression of either an N-terminal region (1–589) or a C-terminal region (590–872) results in dominant negative phenotypes in yeast [27].

Sec10p is one of the subunits that had previously been difficult to produce in a soluble recombinant form. We attempted a number of different strategies to produce soluble Sec10p protein. Initial efforts included the design of twenty different truncations using only secondary structure predictions. The N- and C-termini of these constructs were chosen to reside in non-structured regions so that predicted helices were not disrupted. Truncations were expressed with one of several affinity tags (e.g. His_6_, MBP and GST). The use of MBP as an N-terminal fusion tag appeared promising, as milligrams of soluble Sec10p were produced. However, removal of the MBP tag by proteolytic cleavage resulted in immediate precipitation of Sec10p, suggesting that MBP was solubilizing misfolded and/or aggregated Sec10p protein; indeed, this problem has been previously observed with other proteins [37]. We also tried to co-express Sec10p and several truncations with either its partner exocyst subunit Sec15p [2] or with the chaperones GroEL/GroES [38]. These strategies did not improve the solubility of the Sec10p constructs (data not shown).

**Figure 2:**
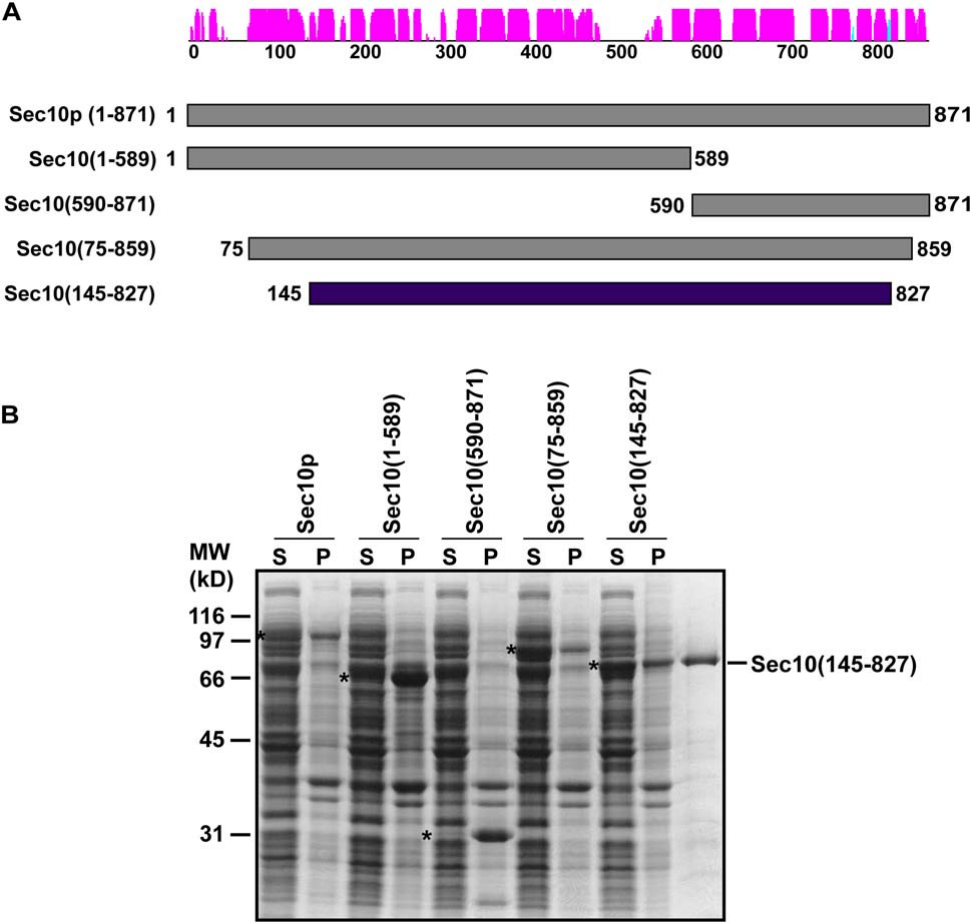
Recombinant Sec10(145–827) is soluble. Several Sec10p truncation constructs designed using secondary structure predictions are not generally soluble. (*A*) Secondary structure prediction [41] and schematic of several representative N- and C-terminal truncations tested. The secondary structure prediction is schematically depicted as in Figure 1. Truncations 1–589 and 590–871 were derived from dominant negative constructs described previously [27]. (*B*) *E. coli* cells were transformed with Sec10p truncation variants cloned with an N-terminal His_6_-tag in the vector pET15b (Novagen). Expression was induced by addition of IPTG to 0.1 mM, and growth was continued at 15°C for 14–18 h. Cells were pelleted, lysed and the insoluble (P) material was separated from the soluble material (S) by centrifugation; these were run on a 10% SDS-PAGE gel and stained with Coomassie blue dye. Asterisks indicate the migration of each construct. For each construct except Sec10(145–827), very little of the His_6_-tagged protein was in the soluble fraction. Although the Sec10(75–859) construct initially appeared promising, it was sticky and aggregated after partial purification on Ni-NTA resin. The right hand lane contains Sec10(145–827) after purification by Ni-NTA resin and gel filtration chromatography.

Based on the HMM structure predictions described above, we identified N- and C-terminal ends of a putative Sec10p structural domain. The HMM analyses predicted a domain with similarity in the N-terminal region to the structure of Exo70p (in the range of 186–465) and in the C-terminal region to the structure of Sec6CT (range of 563–860). Based on this structural assignment, we designed a fragment that would encompass both domains. Consideration of the secondary structure prediction for this region (Figure 2A) led us to clone a construct from residues 145–827, containing a Pro at the N-terminus and a Gly at the C-terminus. This domain was predicted to contain four separate helical bundles, similar to those found in Exo70p and Sec6CT. This construct, Sec10(145–827), was cloned into the T7 expression vector pET15b. We chose to use an N-terminal His_6_ tag in order to maximize the likelihood of obtaining properly folded protein. Our previous experience using other constructs containing His_6_ tags is that this tag is not capable of solubilizing improperly folded recombinant proteins, but is useful for affinity purification. Upon overexpression in BL21(DE3) cells, this 145–827 Sec10p truncation construct was found to be quite soluble, compared to other truncations, including 1–589, 590–871, 75–859, and 55–589 (Figure 2B).

**Figure 3:**
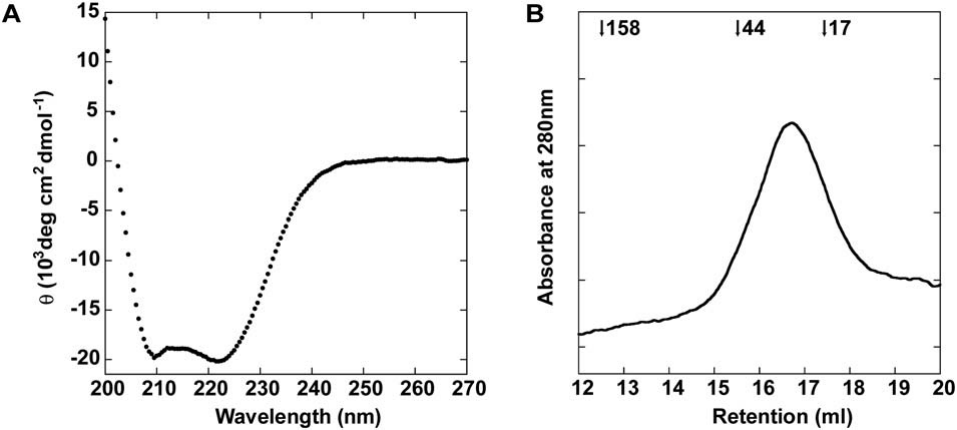
Sec10(145–827) is folded and α-helical. (*A*) The far-UV CD wavelength spectrum of Sec10(145–827) was measured between 200 and 270 nm at 4°C. The characteristic minimum at 222 nm is indicative of ∼60% helicity. (*B*) Gel filtration profile of Sec10(145–827). Purified Sec10(145–827) was applied to a Superdex 200 gel filtration column and the absorbance was monitored at 280 nm. The retention volume of molecular weight standards (in kD) are indicated at the top. Sec10(145–827) elutes in a single, monodisperse peak, although the apparent molecular weight of Sec10(145–827), based on the MW standards, indicates that it elutes slightly smaller than expected, suggesting deviation from a spherical shape, or a small amount of reversible non-specific interaction with the column.

The Sec10(145–827) protein was expressed and purified using Ni-NTA chromatography, followed by size exclusion chromatography to remove several co-purifying contaminants (Figure 2B). Circular dichroism (CD) studies on the purified Sec10(145–827) protein indicate a predominantly helical protein (Figure 3A); calculations of apparent α-helicity suggest that Sec10(145–827) is approximately 60% helical [39], which is consistent with the ∼60% helical content that we predicted. Moreover, purified Sec10(145–827) displays a symmetrical monodisperse gel filtration profile, which indicates a single well-folded protein species (Figure 3B).

### Function of Sec10(145–827)

**Figure 4:**
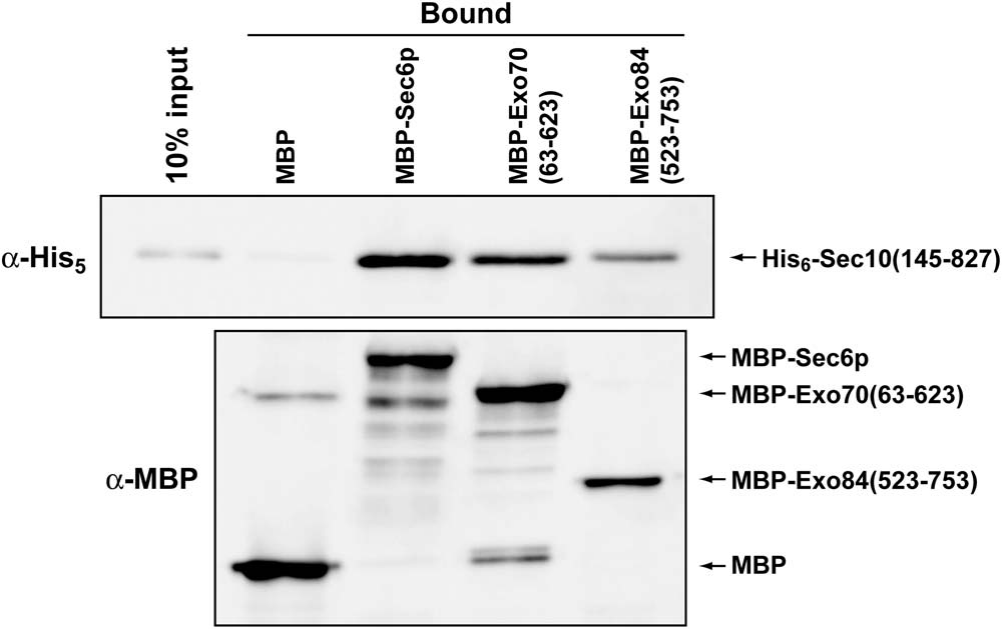
Sec10(145–827) is functional for protein-protein interactions *in vitro*. Sec10(145–827) binds to MBP-Sec6p, MBP-Exo70p (residues 63–623) and MBP-Exo84p (residues 523–753), but not to MBP alone. The MBP, MBP-tagged Sec6p, Exo70p and Exo84p proteins were immobilized on amylose resin and incubated with Sec10(145–827). Equivalent volumes of the bound fractions [10% of the input of Sec10(145–827) is shown in the first lane as a control for the amount of Sec10(145–827) bound] were analyzed on denaturing SDS-PAGE gels. His_6_-tagged Sec10(145–827) and MBP-tagged partners were detected by Western blot analyses using α-His_5_ and α-MBP antibodies, respectively.

Because this construct of Sec10p appeared to be an independently folded structural domain, we tested to see if it was functional. We performed *in vitro* binding experiments with other purified exocyst subunits that bind Sec10p: Sec6p, Exo70p and Exo84p. Sec6p and Exo70p interactions with full-length Sec10p were previously shown by qualitative pull-down binding assays using partially purified proteins [12], [15], while binding of Exo84p had only been observed in yeast two-hybrid assays [28]. For Exo70p (a.a. 63–623) and Exo84CT (a.a. 523–753), we used soluble truncations that had been determined by limited proteolysis and whose structures had previously been determined [12]. Sec6p, Exo70p and Exo84CT were N-terminally tagged with Maltose Binding Protein (MBP) for use in qualitative pull-down experiments. When the purified Sec10(145–827) protein was incubated for 1 h with MBP alone, or with the MBP-tagged proteins, it was found to bind specifically to MBP-Sec6p, -Exo70p and -Exo84CT, above background binding to MBP alone (Figure 4). Therefore, we conclude that Sec10(145–827) is properly folded and contains the binding domain(s) for Sec6p, Exo70p and the C-terminal domain of Exo84p.

Remarkably, under our conditions, only about 10–20% of each recombinant exocyst subunit appears to interact with each other in *in vitro* binding experiments. Similar results have also been observed for the full-length proteins [12], [15]. One possibility for such weak interactions is that only 10–20% of the recombinant proteins are properly folded, although this idea is not supported by our CD and gel filtration results (Figure 3). Alternatively, these experiments were performed at protein concentrations (1–5 µM) that may be substantially lower than the binding constants for the protein-protein interactions. We suggest that the exocyst complex is formed from a combination of many low affinity interactions, which would lead to cooperative assembly and disassembly of the complex at sites of secretion *in vivo*. Regulation of this process by Rho/Rab-GTP binding partners on the vesicle and plasma membranes may trigger conformational changes to activate assembly of the subunits [4].

### Conclusions

We used a profile HMM prediction algorithm to predict that all of the exocyst subunits will have similar helical bundle structures. This approach has been shown to be generally useful to examine other families of proteins without high sequence similarity [40]. The HMM predictions have also proven to be a sensitive computational tool for defining structural domains. It ultimately allowed us to express and purify a soluble domain of the yeast exocyst protein Sec10p that will enable further biochemical and structural analyses. Similar analyses are being used for the other exocyst subunits, which will significantly contribute to our ability to elucidate the structure of the entire exocyst complex and its function in exocytosis.

## Materials and Methods

### Prediction of protein structure homology

We built hidden Markov models for each exocyst component starting from the yeast proteins. Homologous proteins were collected using PSI-Blast for a maximum of 2 iterations with default parameters. Secondary structure was predicted by PSIPRED [41] and added to the profiles. HMMs were built using the HHmake function of the HHpred suite [25]. HMMs were compared to each other and to a PDB HMM database using the HHSearch function of the HHpred suite. Models for each sequence were built using MODELLER [42], and evaluated for the general trend of atomic interaction by statistical potential [43] and a composite model evaluation criterion [44].

To create a soluble construct of Sec10p, the HMM profile of Sec10p was directly compared with the profiles for the known structures of Exo70p, Sec15CT, Sec6CT and Exo84CT. A structural domain in the range of residues 148–867 was predicted. Using this information and secondary structure predictions (http://npsa-pbil.ibcp.fr/ and [41], we chose the N- and C-terminal ends of the structural domain to be residues 145 and 827, residues predicted to be at the ends of helices.

### Protein Expression and Purification

Genes encoding full-length Sec10p (residues 1–872), the various truncations [Sec10(1–589); Sec10(590–871); Sec10(75–859), and the predicted structural domain Sec10(145–827)] were amplified by polymerase chain reaction and cloned into the *Nde*I and *Bam*HI restriction sites of the vector pET15b (Novagen), which introduces a 6-histidine tag (His_6_) at the N termini. All constructs were confirmed by sequencing. All proteins were expressed in *E. coli* BL21(DE3) cells. To maximize protein solubility, cells were grown in LB to an OD at 600 nm of ∼0.4 at 37°C. Cells were shifted to 15°C until the OD_600_ reached between 0.6–0.9. Protein expression was induced with 0.1 mM isopropyl-β-D-thiogalactoside (IPTG), and cells were grown for an additional 14–18 h at 15°C. Cells were harvested and frozen at −80°C until lysis. His_6_-tagged Sec10(145–827) protein was purified using nickel-NTA resin (Qiagen). β-Mercaptoethanol (5 mM) or dithiothreitol (DTT, 1 mM) was used in all buffers. Sec10(145–827) was purified using a Superdex 200 16/60 gel filtration column (GE) in KPhos buffer (10 mM potassium phosphate at pH 7.4 containing 140 mM KCl and 1 mM DTT) plus 10% glycerol. Fractions were analyzed by SDS-PAGE and stained with Coomassie blue. Those fractions containing >90% pure protein were pooled. Protein was concentrated using a stirred cell concentrator (Millipore) to ∼1 mg/ml, and the protein concentration was determined by measuring the absorbance at 280 nm and by a quantitative ninhydrin protein assay [45]. Protein was flash frozen in liquid nitrogen in KPhos buffer containing 10% glycerol and stored at −80°C.

### Circular Dichroism Spectroscopy

CD spectra were recorded on a J810 spectropolarimeter (Jasco) fitted with a Peltier-type temperature controller set to 4°C. The Sec10(145–827) protein was at a concentration of 0.8 µM in KPhos buffer containing 1 mM DTT and 10% glycerol. The spectra were recorded as an average of three scans from 200 to 270 nm, in a 1 mm path-length quartz cuvette (Hellma). For each spectrum, the minimum at 222 nm was used to estimate the mean residue ellipticity and percent helicity [39].

### Analytical Gel Filtration

Sec10(145–827) was chromatographed on a Superdex 200 10/30 column (GE) at a protein concentration of 1 µM. The column was pre-equilibrated in sodium phosphate buffer containing 300 mM NaCl, 10% glyercol and 1 mM DTT; eluted peaks were observed by monitoring the absorbance at 280 nm. The gel filtration column was calibrated using standards (thyroglobulin, 670 kD; γ-globulin, 158 kD; ovalbumin, 44 kD; myoglobin, 17 kD; Bio-Rad).

### MBP pull-down assays and Western blot analyses

Sec6p (residues 1–805), Exo70p (residues 63–623; [12], and Exo84CT (residues 523–753; [12] were subcloned into pMALc2X (New England Biolabs). Maltose binding protein (MBP) was expressed at 37°C and MBP-tagged Sec6p, Exo70p, and Exo84CT were expressed at 20°C for 3 h after induction with 0.1 mM IPTG. They were purified using amylose resin affinity chromatography (New England Biolabs). The binding reactions contained purified MBP-tagged proteins immobilized on amylose resin in binding buffer (10 mM HEPES pH 7, 100 mM NaCl, 0.1% NP-40, and 1 mM DTT), and an equimolar amount of the purified Sec10(145–827) protein (1 µM) was added. The reactions were incubated for 1 h at 4°C with mixing to allow binding. Beads were centrifuged and washed three times in binding buffer and analyzed by SDS-PAGE. Proteins were transferred to nitrocellulose and probed with α-His_5_ (Qiagen) or α-MBP antibodies (Invitrogen). Western blots were developed using horseradish peroxidase-conjugated α-mouse IgG (Roche), followed by chemiluminescent detection (ECL; Amersham) and luminescent image analysis (FUJIFILM LAS-3000). The blots shown are representative of at least three separate experiments.
